# High-Iron Bauxite Residue (Red Mud) Valorization Using Hydrochemical Conversion of Goethite to Magnetite

**DOI:** 10.3390/ma15238423

**Published:** 2022-11-26

**Authors:** Andrei Shoppert, Dmitry Valeev, Mamodou Malal Diallo, Irina Loginova, Marie Constance Beavogui, Abdukhakim Rakhmonov, Yevgeniy Ovchenkov, Denis Pankratov

**Affiliations:** 1 Department of Non-Ferrous Metals Metallurgy, Ural Federal University, 620002 Yekaterinburg, Russia; 2 Laboratory of Advanced Technologies in Non-Ferrous and Ferrous Metals Raw Materials Processing, Ural Federal University, 620002 Yekaterinburg, Russia; 3 Laboratory of Sorption Methods, Vernadsky Institute of Geochemistry and Analytical Chemistry of the Russian Academy of Sciences, 119991 Moscow, Russia; 4 Laboratoire de Recherche Appliquée en Géoscience et Environnement, Institut Supérieur des Mines et Géologie de Boké (ISMGB), Boke 120, Guinea; 5 Department of Physics, Lomonosov Moscow State University, 119991 Moscow, Russia; 6 Department of Chemistry, Lomonosov Moscow State University, 119991 Moscow, Russia

**Keywords:** alkali leaching, red mud, high-iron waste, disposal, Al-goethite, conversion, magnetite

## Abstract

Bauxite residue (BR), also known as red mud, is a byproduct of the alumina production using the Bayer process. This material is not used to make iron or other iron-containing products worldwide, owing to its high content of sodium oxide and other impurities. In this study, we investigated the hydrochemical conversion of goethite (FeOOH) to magnetite (Fe_3_O_4_) in high-iron BR from the Friguia alumina refinery (Guinea) by Fe^2+^ ions in highly concentrated alkaline media. The simultaneous extraction of Al and Na made it possible to obtain a product containing more than 96% Fe_3_O_4_. The results show that the magnetization of Al-goethite and Al-hematite accelerates the dissolution of the Al from the iron mineral solid matrix and from the desilication product (DSP). After ferrous sulfate (FeSO_4_·7H_2_O) was added directly at an FeO:Fe_2_O_3_ molar ratio of 1:1 at 120 °C for 150 min in solution with the 360 g L^−1^ Na_2_O concentration, the alumina extraction ratio reached 96.27% for the coarse bauxite residue size fraction (Sands) and 87.06% for fine BR obtained from red mud. The grade of iron (total iron in the form of iron elements) in the residue can be increased to 69.55% for sands and 58.31% for BR. The solid residues obtained after leaching were studied by XRD, XRF, TG-DTA, VSM, Mössbauer spectroscopy, and SEM to evaluate the conversion and leaching mechanisms, as well as the recovery ratio of Al from various minerals. The iron-rich residues can be used in the steel industry or as a pigment.

## 1. Introduction

BR is a solid fraction of red mud. Each year, more than 150 million tons of red mud are produced during the Bayer process, which is used to extract Al from bauxites by the alkaline method [1]. As a result of this method, red mud has high contents of caustic alkali and other impurities, which makes it very toxic [2]. Moreover, the concentration of heavy metals in BR can be doubled after alumina extraction [3,4]. The utilization rate of BR is very low, owing to its high toxicity and corrosivity [5,6,7].

BR contains a number of valuable components, such as iron, rare earth elements (REEs), vanadium, titanium, etc. [8,9,10,11,12,13,14]. The extraction of these elements from BR, together with a reduction in its toxicity, can be economically and ecologically beneficial. For example, BR produced from the Fria bauxite deposit in Guinea has very high iron contents; 40% of the iron in red mud obtained after digestion of bauxites in the Friguia alumina refinery or 55–60% of the iron in the sands obtained by the gravity method of separation of BR before the thickening and washing step [15].

Various methods of iron production from BR have been studied to date. These methods can be divided into pyro- and hydrometallurgical methods [16]. Pyrometallurgical methods include magnetic separation after preliminary reductive roasting [17], reductive sintering with various fluxes [18,19], and smelting of BR with a reducing agent to produce pig iron [20,21]. The major disadvantage of these methods is their high energy consumption, as the temperatures of these processes can reach as high as 1000–1750 °C [22].

Li et al. [23,24] and Pasechnik et al. [25] showed that hematite from BR can be converted into magnetite during a hydrothermal reduction in the presence of iron powder, Fe^2+^, and OH^−^ ions (Equations (1)–(3)). However, a high-pressure process with a leaching temperature of more than 250 °C is required for complete iron conversion into magnetite.

Fe + H_2_O + OH^−^ = HFeO_2_^−^ + H_2_, (1)

Fe^2+^ + 3OH^−^ = HFeO_2_^−^ + H_2_O, (2)

Fe_2_O_3_ + HFeO_2_^−^ = Fe_3_O_4_ + OH^−^. (3)

Furthermore, Zhou et al. [26] showed that the addition of Al during high-temperature digestion of high-iron bauxite not only transforms iron into hematite covered by magnetite but also accelerates Al extraction from Al-goethite, which is difficult to dissolve by the Bayer method. However, high-pressure leaching at 270 °C is required for the formation of iron-enriched residue with an iron content of 56%. However, iron content remains low, especially if pigment-quality magnetite is proposed as a final product.

Vu et al. [27] and Bohacek et al. [28] proposed a method for the production of black pigment-quality magnetite from jarosite (NH_4_Fe_3_(SO_4_)_2_(OH)_6_) by decomposition in an ammonia or sodium alkali environment in the presence of ferrous sulfate (Equations (4) and (5)). The mixture then heated to 90 °C and maintained for 7 h to obtain a well-crystallized product with iron content of more than 70% [27].

NH_4_Fe_3_(SO_4_)_2_(OH)_6_ + 2OH^−^ → NH_4_^+^ + 3Fe(OH)_3_ + 2SO_4_^2−^, (4)

2Fe(OH)_3_ + Fe^2+^ + 2OH^−^ → Fe_3_O_4_ + 4H_2_O, (5)

This freshly precipitated Fe(OH)_3_ is very reactive, so magnetite can be formed at a lower temperature (90 °C vs. 250 °C for hematite in bauxites). Hage et al. [29] suggested a method to produce magnetite from jarosite using MgO as a neutralizing agent and acid-treated cellulose as a reductant. Using a reducing agent saves expensive alkali, which is neutralized and turned into Na_2_SO_4_ by adding FeSO_4_. Both methods allow for the obtainment of high-quality magnetite products from jarosite, which itself contains few impurities.

In this work, a novel method of atmospheric pressure leaching (APL) of Guineas high-iron BR in the presence of FeSO_4_ was proposed, which led to almost complete dissolution of Al from the solid matrix of the sands with the formation of pigment-quality magnetite containing about 70% Fe. The amount of Al extracted from red mud was also higher than 87%; however, the iron content in the solid residue reached only 58%. The optimum conditions of alumina extraction and iron mineral conversion, as well as the mechanism of these processes, were verified. The solids were examined using X-ray diffraction (XRD), X-ray fluorescence (XRF), scanning electron microscopy with energy-dispersive spectroscopy (SEM-EDS), Mossbauer spectrometry, vibrating sample magnetization (VSM), and spectrophotometry methods.

## 2. Materials and Methods

### 2.1. Chemical Composition of Samples

The chemical composition of the raw BR and residues after leaching was analyzed using powder X-ray fluorescence spectrometry with an Axios MAX spectrometer (Malvern Panalytical Ltd., Almelo, The Netherlands). Tablet-shaped samples (Ø 20 mm; 300 mg) were prepared for analysis via pressing, and polystyrene was used as a binder at a ratio of 5:1. Super Q software was used to calculate the metallic contents of the samples.

The Sc content in BR and sands leaching residues were determined by inductively coupled plasma mass spectrometry (ICP-MS) using an X Series II quadrupole mass spectrometer (Thermo Scientific, Dreieich, Germany). Quantitative determination of the Sc was carried out using iPlasmaProQuad software (GEOKHI RAS, Moscow, Russia).

### 2.2. Phisical Properties of Samples

The phase composition of the raw BR and residues after leaching was analyzed by X-ray diffraction with a Difrei-401 diffractometer (JSC Scientific Instruments, Saint Petersburg, Russia) and a Cr-Kα radiator with 2θ angles ranging from 15° to 140°. The X-ray source operated with an output of 25 kW and an exposure time of 30 min. The diffraction data were processed using Match 3! software (Crystal Impact GmbH, Bohn, Germany). The particle size distribution of the samples was measured with a SALD-2201 laser diffraction particle size analyzer (Shimadzu, Kyoto, Japan). The surface morphology and elemental mapping of the raw materials and the residues were investigated using SEM–EDX methods on a Vega III microscope (Tescan, Brno, Czech Republic). The Mössbauer analysis of raw BR and sand residues after leaching was performed using an MS1104EM spectrometer (Cordon, Rostov-on-Don, Russia) at 296 ± 3 K and 77.6 ± 0.5 K temperatures in a vacuum cryostat. ^57^Co nuclei in an Rh matrix with 3 mCi activity (RITVERC, Saint Petersburg, Russia) were used as the γ-radiation source. SpectrRelax 2.8 software (Lomonosov Moscow State University, Moscow, Russia) was used to analyze the Mössbauer spectra. Values of chemical shifts are presented relative to α-Fe. Magnetic measurements were performed using an MPMS-XL-7 SQUID magnetometer (Quantum Design, San Diego, CA, USA). TG-DTA analysis was performed using an STA 449 F3 Jupiter simultaneous thermogravimetry analyzer (Netzsch, Selb, Germany) in the range of 33–1100 °C at a heating rate of 10 °C min^−1^. An air atmosphere was used, with a flow rate of 20 mL min^−1^.

### 2.3. Materials

**Figure 1:**
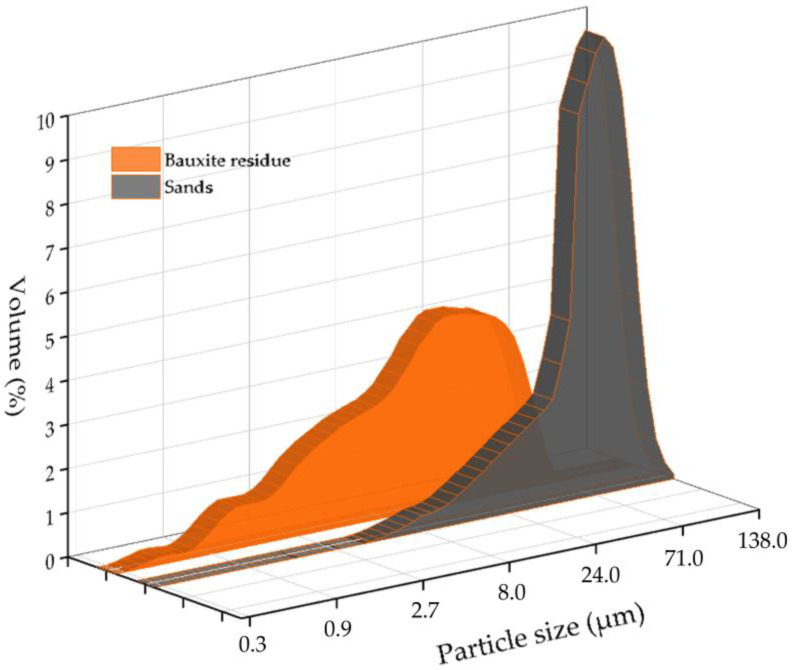
The particle size distribution of the bauxite residue (BR) and sands from the Friguia alumina refinery.

**Figure 2:**
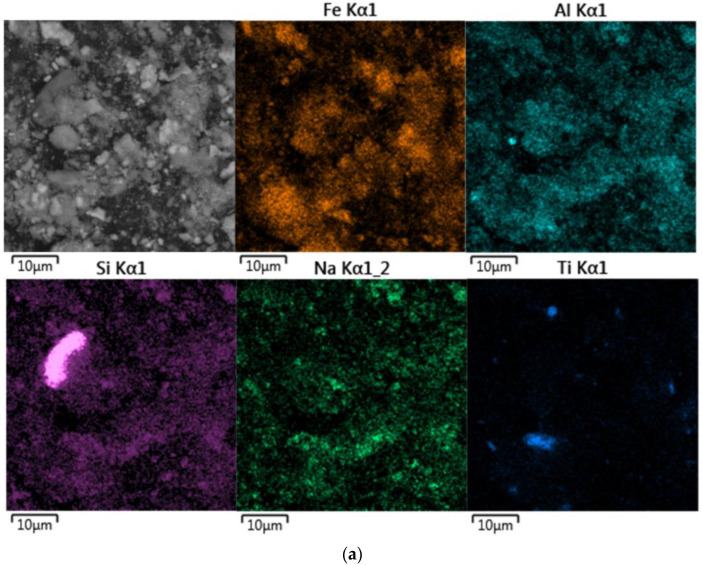

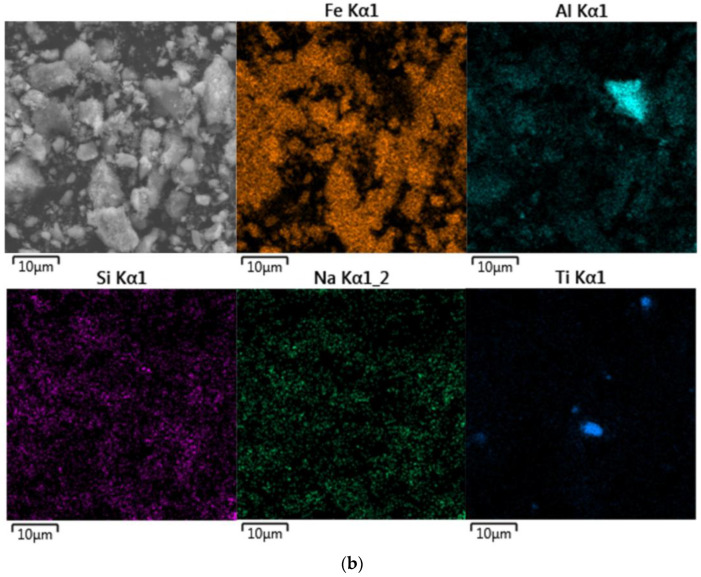
Backscattered electron (BSE) image and elemental mapping of BR particle surface (**a**); BSE images and elemental mapping of the sand particle surface (**b**).

The bauxite residue and sands used in this research were obtained from the Friguia alumina refinery in Guinea with GPS coordinates 10.39° N, 13.58° W. Sands are the coarser particles of residue obtained from red mud by separation through hydrocyclones before the thickening and washing step. The chemical compositions of BR and the sands are presented in Table 1. Figure 1 shows the particle size distribution of samples. Sands obtained using a hydrocyclone separator are significantly coarser than BR, and the particle size distribution of the sands is more uniform. Figure 2 shows the morphology and elemental mapping of the samples. BR is composed of particles smaller than 10 μm, and the elemental mapping of Fe, Na, Si, and Al are similar, which can be explained by the precipitation of DSP and gibbsite during the thickening and washing steps. The elemental map of the sands shows the presence of many goethite particles and some single aluminum particles with a small amount of silicon and sodium and a considerable amount of iron on the surface, implying that desilication of the solution ends during the thickening and washing stage. Reprecipitation of gibbsite from a diluted aluminate solution during the washing step also leads to an increased concentration of aluminum in the BR. 

The other reagents, NaOH and FeSO_4_·7H_2_O, were of analytical grade. Sodium alkaline solutions with a Na_2_O concentration of 330–400 g L^−1^ were obtained by mixing NaOH with distilled water.

### 2.4. Experimental

The extraction of Al with NaOH and the conversion of goethite to magnetite were carried out in a 0.5 L thermostated stainless steel reactor. The reactor has openings for stirring, as well as for temperature control and recycling of evaporated water through a water-cooled condenser. The L:S ratio and the stirring speed in all experiments were 100 g L^−1^ and 300 rpm, respectively. The molar ratio of Fe^2+^ added in the form of iron sulphate to Fe_2_O_3_ in BR or sands according to Equation (3) was equal to 1.0. The BR, sands, and iron sulphate were added to a hot solution with a Na_2_O concentration of 330 g L^−1^ to 400 g L^−1^. This made it possible to perform the atmospheric leaching process at a temperature of 120 °C. The temperature of the leaching was varied from 100 to 120 °C, and the leaching time was varied from 1 to 5 h. After leaching, the pulp was filtered, and the solid residue was dried at 110 °C for 8 h before analysis.

### 2.5. Experimental Data Calculation

In order to avoid the mutual influence of factors and to reduce the number of experiments, a Box–Benken experimental design created using Statistica 13 software (TIBCO, Hamburg, Germany) was used in this research. The design consists of three blocks of fifteen experiments each, with varying parameters at three levels. The output parameters are the extraction of aluminum into solution and the concentration of iron in the solid residue.

A statistically based automated neural network (SANN) was used to model the Al and Fe extraction and Na_2_O concentration in the solid residue. SANN is an artificial-intelligence-based method that adjusts the result of modeling until the desired quality is obtained. Statistica 13 was used to make SANN models using a multilayer perceptron (MLP) method.

The amount of Al extracted from the various aluminum minerals (X) into the solution was estimated using Equation (6):

X = (m_1_ × X_1_ − m_2_ × X_2_)/(m_1_ × X_1_), (6)

where m_1_ is the mass of the original sample (g), X_1_ is the mineral content in the original sample (%), m_2_ is the mass of the leaching residue (g), and X_2_ is the mineral content in the leaching residue (%).

To assess the content of Na in the raw materials and solid residue, we assumed that all the Na in the product is represented by DSP Equation (7):

DSP = [(Na × 23)/(27 × Al)] × 100%,(7)

where Na is the content of sodium in a sample (%), Al is the Al content in a sample (%), and 23/27 is the molar ratio of Na vs. Al in the sodalite.

In the case of sands that have only two Al minerals, Equation (8) was used to calculate the aluminum contained in the solid matrix:

Solid matrix_sands_ = [(Al − DSP)/Al] × 100%, (8)

where Al is the Al content [20] in a sample (%), and DSP is the amount of Al in the DSP in the sample calculated by Equation (7) (%).

To determine the amount of aluminum hydroxides in the solid matrix of BR iron minerals, we assumed that the amount of this Al has a linear dependence on the Fe content in the residue (Equation (9)):

Solid matrix_BR_ = [(Solid matrix_sands_ × Fe_BR_)/Fe_sands_] × 100%, (9)

where Solid matrix_sands_ is the amount of Al in the solid matrix of the raw sand iron minerals calculated by Equation (8) (%), Fe_BR_ is the Fe content in the BR, and Fe_sands_ is the Fe content in the raw sands (%).

Therefore, all the remaining Al in the BR is represented by reprecipitated gibbsite.

Blackness (jetness) evaluation was conducted using an SR-60 colorimeter (Shenzhen ThreeNH Technology Co., Shenzhen, China). To obtain a single numerical result, the calculation of the magnitude of the blackness (My) was based on one of the values of the color coordinates, i.e., L (brightness), which was determined by Equation (10):

My = 100 × log(100/L).(10)

This only determines how light or dark the sample is without taking the color shade into account. Because the shade of black affects how dark something looks, the degree of jetness (Mc) was determined by converting CIE LAB results to CMYK in the color model with the following Equation (11):

M_c_ = 100 × ((log(X_n_/X) − log(Y_n_/Y) + log(Z_n_/Z)). (11)

where the Mc value describes a higher blackness if the shade is blue (Z) and a lower saturation if the shade is yellow (Y), and X_n_, Y_n_, and Z_n_ are the standard color values of the illuminating light type.

## 3. Results and Discussion

### 3.1. The Nature of the Al-Containing Phase in the Raw Materials

**Figure 3:**
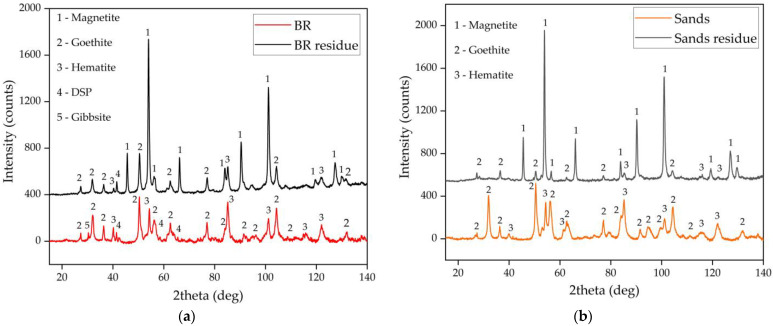
The XRD patterns of the BR and the solid residue obtained after BR leaching under optimal conditions (**a**) and the XRD patterns of the sands and the solid residue obtained after sand leaching under optimal conditions (**b**).

Previous research by Zhou et al. [26] showed that the rate of Al extraction from Guinea bauxite with a high iron content is limited by the presence of aluminum-substituted goethite (Al-goethite), which is refractory using conventional Bayer process conditions. To study the occurrence of Al in the BR and the sands from the Friguia alumina refinery, the phase composition of the samples was studied using XRD, TGA, and Mössbauer spectrometry. The XRD patterns of the BR and the sands are shown in Figure 3. Figure 3a,b, indicating differences in hematite, goethite, and gibbsite content in the raw BR and sands. The peaks of goethite are higher in the sand samples, whereas hematite is found in higher concentrations in the BR. There are also peaks of sodalite (DSP) in the BR that are not observed in the sands because the desilication of the solution in the refinery takes place in the thickening and washing step of BR after sand separation.

**Figure 4:**
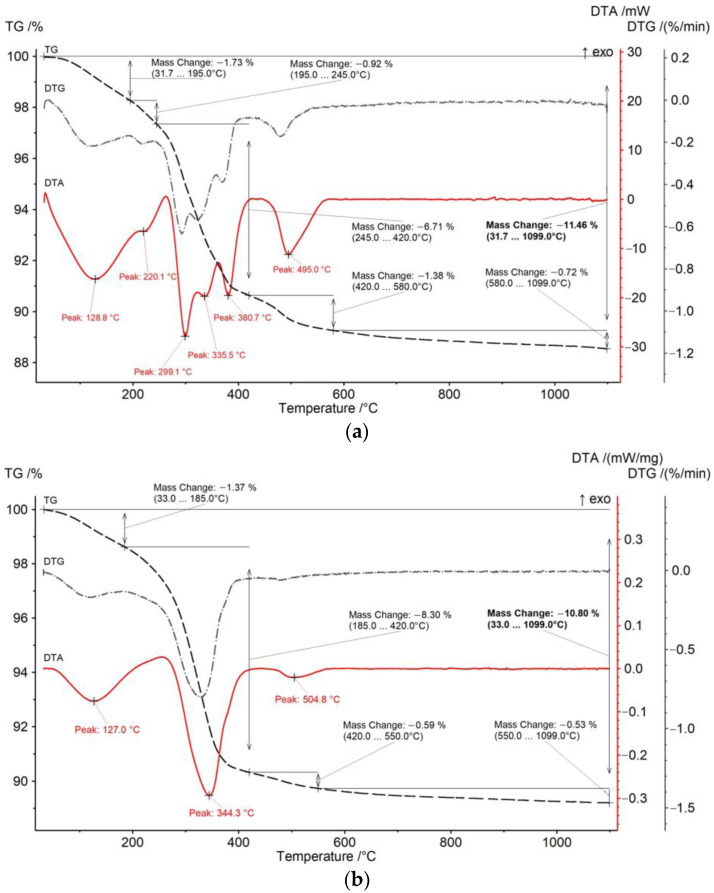
TG-DTA curves of the BR (**a**) and sands (**b**) at varying temperatures.

Figure 4 shows the TG-DTA curves of the BR and the sands. There are significant differences in the DTA of these two samples. Figure 4a shows that the decomposition of BR occurred in five stages, similar to the results obtained by Zhou et al. [30] for Al-goethite containing bauxite residues. The first stage (from 25 to 220 °C) is attributed to the loss of moisture [21]. The second stage (from 220 to 330 °C) with an endothermic peak at 299.1 °C is associated with the decomposition of gibbsite [31]. The third stage occurred at temperatures between 330 and 370 °C, with an endothermic peak of goethite and Al-goethite decomposition observed at 335.5 °C [32]. Between 335 and 420 °C, in the fourth stage, the boehmite undergoes decomposition, followed by the fifth stage with an endothermic peak of hydrosodalite decomposition at 495 °C [33]. The TG-DTA curve of the sands shows only endothermic peaks of goethite at 344.3 °C and a much smaller peak of hydrosodalite at 504.8 °C. These findings indicate that there is practically no gibbsite in the sands and that all aluminum is enclosed in a solid matrix of minerals and DSP.

**Figure 5:**
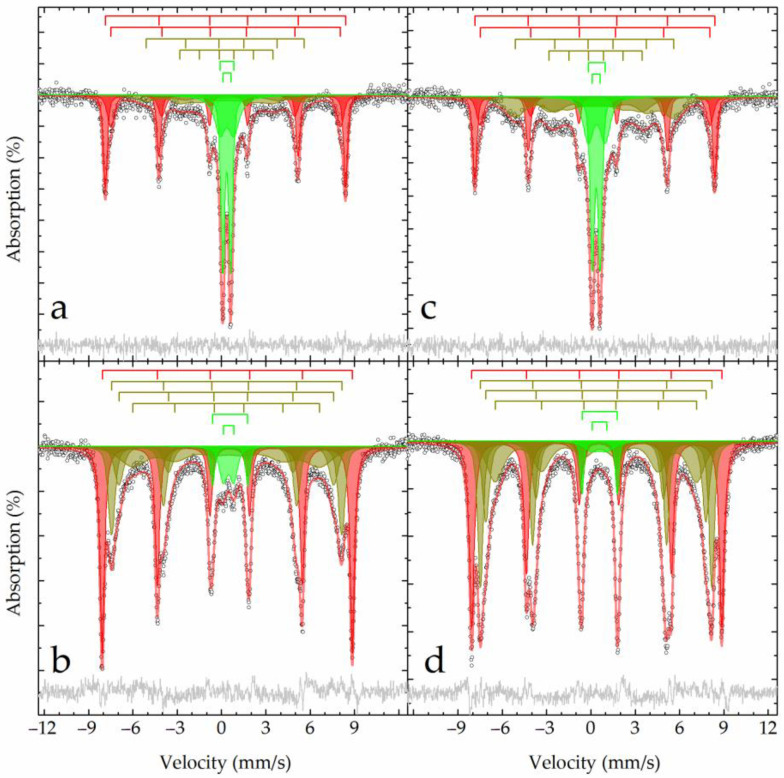
Mössbauer spectra of the BR (**a**,**b**) and sand (**c**,**d**) samples obtained at 296 K (**a**,**c**) and 77.6 K (**b**,**d**) and their model description according to Table 2.

Figure 5 shows the results of the Mössbauer analysis of raw BR and sands. Samples at room temperature have a similar profile, comprising combinations of an intense paramagnetic doublet and a sextet with fairly narrow resonance lines (Figure 5). A noticeable difference between the spectrum of the sands sample and BR is that the first sample has extended absorption between the first and fifth lines of the sextet (Figure 5a,c). At the boiling point of nitrogen, the shape of the spectrum changes significantly; the doublet almost completely disappears, and the outer lines of the narrow sextet split in pairs (Figure 5b,d), supporting the complex composition of the materials.

**Table 2:** The subspectrum parameters describing the experimental Mössbauer spectra obtained at varying temperatures for BR and sand samples.

Sample			BR					Sands				
Temperature, K	Phase	№	δ	ε (Δ = 2ε)	Γ_exp_	H_eff_	S	δ	ε (Δ = 2ε)	Γ_exp_	H_eff_	S
			mm/s			kOe	%	mm/s			kOe	%
296	α-(Fe_1−x_Al_x_)_2_O_3_	1	0.37 ± 0.01	−0.10 ± 0.01	0.34 ± 0.01	504.7 ± 0.2	26 ± 1	0.37 ± 0.01	−0.10 ± 0.01	0.36 ± 0.01	504.1 ± 0.3	19 ± 1
		2	0.37 ± 0.01	−0.09 ± 0.01	0.59 ± 0.03	482 ± 1	15 ± 1	0.36 ± 0.01	−0.08 ± 0.01	0.58 ± 0.04	482 ± 2	11 ± 1
	α-Fe_1−x_Al_x_OOH	3	0.45 ± 0.01	−0.21 ± 0.01	1.60 ± 0.01	332.1 ± 0.1	10 ± 1	0.45 ± 0.05	−0.21 ± 0.05	1.60 ± 0.09	332 ± 2	23 ± 1
		4	0.32 ± 0.01	−0.01 ± 0.01	1.60 ± 0.01	195.5 ± 0.1	11 ± 2	0.32 ± 0.07	−0.01 ± 0.05	1.60 ± 0.09	196 ± 4	15 ± 1
	β-Fe_1−x_Al_x_O(OH, Cl)	5	0.37 ± 0.01	(1.0 ± 0.3)	0.88 ± 0.07		15 ± 5	0.39 ± 0.01	(1.1 ± 0.1)	0.84 ± 0.09		12 ± 2
		6	0.36 ± 0.01	(0.53 ± 0.01)	0.33 ± 0.03		23 ± 5	0.36 ± 0.01	(0.52 ± 0.01)	0.36 ± 0.01		19 ± 2
77.6	α-(Fe_1−x_Al_x_)_2_O_3_	1	0.48 ± 0.01	−0.08 ± 0.01	0.37 ± 0.01	525.2 ± 0.1	38.4 ± 0.7	0.47 ± 0.01	−0.07 ± 0.01	0.40 ± 0.01	525.1 ± 0.1	31.8 ± 0.5
	α-Fe_1−x_Al_x_OOH	2	0.47 ± 0.01	−0.12 ± 0.01	0.56 ± 0.01	482.9 ± 0.4	22 ± 1	0.47 ± 0.01	−0.12 ± 0.01	0.43 ± 0.01	485.5 ± 0.2	24.9 ± 0.9
		3	0.47 ± 0.01	−0.15 ± 0.01	0.75 ± 0.04	451 ± 1	13 ± 1	0.47 ± 0.01	−0.13 ± 0.01	0.56 ± 0.02	461.8 ± 0.5	18 ± 1
	β-Fe_1−x_Al_x_O(OH, Cl)	4	0.40 ± 0.04	−0.09 ± 0.03	2.18 ± 0.01	391.6 ± 0.1	19.0 ± 0.5	0.47 ± 0.01	−0.14 ± 0.01	1.34 ± 0.04	422 ± 2	21 ± 1
	Fe^3 +^_Oh_ (DSP)	5	0.59 ± 0.01	(2.37 ± 0.01)	0.33 ± 0.02		2.9 ± 0.1	0.59 ± 0.01	(2.38 ± 0.01)	0.25 ± 0.01		2.5 ± 0.2
		6	0.47 ± 0.01	(0.71 ± 0.02)	0.62 ± 0.03		4.8 ± 0.2	0.57 ± 0.05	(1.0 ± 0.1)	1.2 ± 0.3		2.0 ± 0.3

δ—isomer shift; ε (Δ)—quadrupole shift (splitting); Γ_exp_—line width; H_eff_—hyperfine magnetic field; S—relative area of a subspectrum №.

The Mössbauer spectra of the raw materials can be described by a superposition of four sextets and two doublets (Figure 5, Table 2). Two outer sextets at 296 K and one outer sextet at 78 K correspond to aluminum-substituted hematite—α-(Fe_1−x_Al_x_)_2_O_3_ [34]. The low value of magnetic splitting, even for frozen samples, and the absence of a Morin transition (TM = 260 K for α-Fe_2_O_3_) prevent us from attributing these subspectra to pure, unsubstituted hematite—α-Fe_2_O_3_ [22,35]. The isomorphic substitution of aluminum for iron atoms [36] leads to a decrease in the effective magnetic field recorded in the spectra by approximately 1 kOe by 1 w.% for 300 K [37,38]. According to an analysis of the data presented in [38], the degree of substitution (x) in alumina hematite—α-(Fe_1−x_Al_x_)_2_O_3_ can be expressed in terms of the effective magnetic field at room temperature as:

x = 5.4982 − 0.0107 × Heff,(12)

Based on the experimental data (Table 2) for the studied samples using Equation (12), it is possible to determine the composition of Al-hematite as α-(Fe_0.9_Al_0.1_)_2_O_3_ for the first subspectrum and as α-(Fe_0.7_Al_0.3_)_2_O_3_ for the second subspectrum (Table 2).

The next pair of sextets poorly resolved at room temperature, upon cooling to the boiling point of nitrogen, transforms into a pair of well-defined sextets with parameters close to those of goethite [39]. The reason for the underestimated value of the effective magnetic field, even at low temperatures (whereas for true goethite, it can reach 507 kOe [16]), is the partial isomorphic substitution of iron atoms by diamagnetic aluminum atoms [36]. Accordingly, we can assert the formation of Al-goethite—α-Fe_1−x_Al_x_OOH, for which similar temperature transformations of the spectrum are discussed in detail in [16].

The paramagnetic part of the high-temperature Mössbauer spectra may have a dual nature. On the one hand, 7–10% of the area of this pair of doublets can be related to the superparamagnetic fractions of the Al-hematite and Al-goethite described above [40]. The remaining part, as shown in [16], refers to akageneite substituted by aluminum—β-Fe_1−x_Al_x_O(OH, Cl), which, when cooled to the boiling point of nitrogen, transforms into a broadened sextet (Table 2). The remaining minor components in the low-temperature spectra in the form of doublets correspond to Fe^3+^ ions in a high-spin state and octahedral oxygen environment [41], which can isomorphically replace Al^3+^ ions [36] in the crystal lattice of, for example, sodalite [42].

Based on the chemical analysis and the phase composition of the raw materials, the distribution of aluminum by minerals was calculated. Table 3 shows the results of the semiquantitative calculation of the aluminum-containing phase composition.

### 3.2. The Effect of Leaching Parameters on the Al Extraction and the Fe, Na Content in the Solid Residue

**Figure 6:**
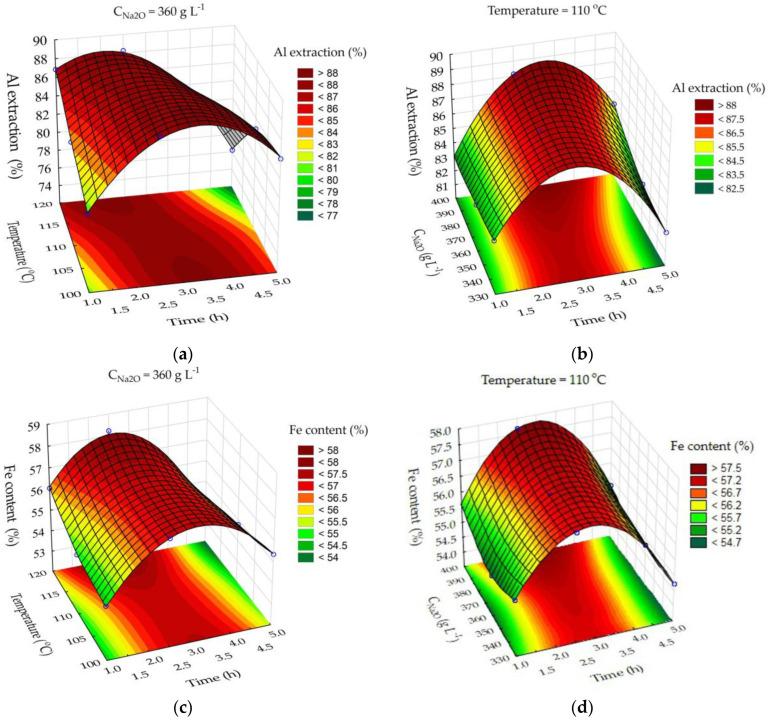

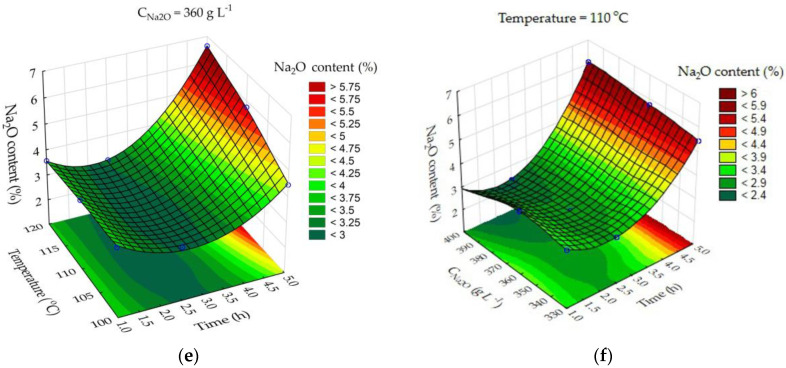
Neural network response surfaces for the effect of time and temperature on Al extraction from bauxite residue (BR) (**a**); effect of time and Na_2_O concentration on Al extraction from BR (**b**); effect of time and temperature on Fe content in the solid residue (**c**); effect of time and Na_2_O concentration on Fe content in the solid residue (**d**); effect of time and temperature on Na_2_O content in the solid residue (**e**); effect of time and Na_2_O concentration on Na_2_O content in the solid residue (**f**).

To determine the effect of leaching parameters on the rate of Al extraction and the Fe, Na_2_O content in the solid residue, experiments were performed according to the method described in Section 2.5. A neural network model of the process was obtained based on the experimental results. Figure 6 shows the response surfaces of the model constructed by varying time, temperature, and the Na_2_O concentration of the solution in the leaching process of the BR.

**Figure 7:**
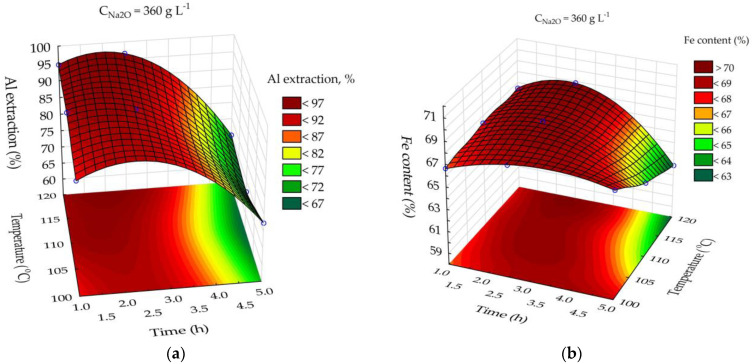

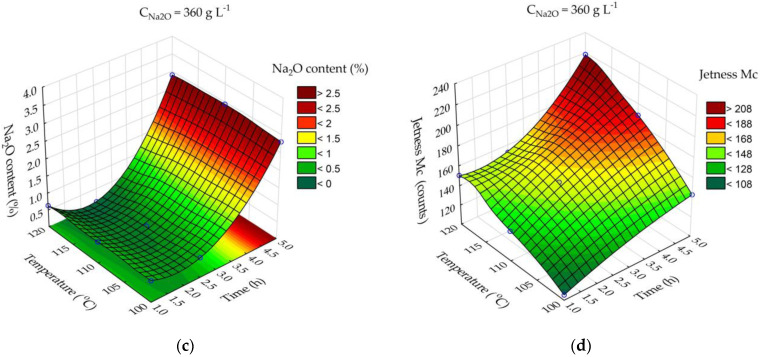
Neural network response surfaces for effect of time and temperature on Al extraction from sands (**a**); effect of time and temperature on Fe content in the sand residue (**b**); effect of time and temperature on Na_2_O content in the sand residue (**c**); effect of time and temperature on the jetness (**d**).

The major effect on Al extraction degree (Figure 6a,b) is caused by leaching time and temperature. Figure 6a shows that the Al extraction degree increases for the first 2.0–3.5 h of leaching. After 3.5 h at 100 °C and 3 h at 110 °C, Al begins to precipitate in the form of desilication product (DSP), which can be confirmed by the increased content of Na_2_O in the solid residue after 4 h of leaching (Figure 6e,f). After 4 h of leaching, the iron content in the solid residue decreases as a result of DSP precipitation (Figure 6c,d). Nevertheless, a high Al extraction degree was achieved after 2 h at all leaching temperatures—more than 80%. After 2.5 h of leaching at T = 120 °C, the total Fe content in the solid residue can be increased from 41.7% (Table 1) to 56%. The Na_2_O concentration in the solution did not have a significant effect on the Al extraction or the Fe, Na_2_O content in the solid residues (Figure 6b,d,f). Therefore, the effect of Na_2_O concentration in the solution on sand leaching is not shown (Figure 7).

As shown in Figure 6a, the major effect on Al extraction is caused by leaching time. After 2 h of leaching at T = 120 °C and C_Na2O_ = 360 g L^−1^, the Al extraction degree was higher than 95%. After 2–2.5 h of leaching, the solid residue had the highest Fe content (Figure 7b). Therefore, the optimal leaching parameters for sands and BR are T = 120 °C, C_Na2O_ = 360 g L^−1^, and leaching time = 2.5 h. The content of Fe in the sand solid residue under these conditions was about 70%, whereas the Na_2_O content was less than 0.25% (Figure 7c); therefore, this product can be used as a pigment-quality magnetite.

One of the most important characteristics of pigments is their jetness. Figure 7d illustrates how leaching time and temperature affect the jetness of the magnetite concentrate obtained after the leaching of sands in the presence of Fe^2+^. According to these data, 5 h of leaching is necessary to achieve a jetness of more than 200. The jetness of modern pigments can be more than 300 [43]. Therefore, there Is a dilemma in achieving both high jetness and low impurities because after 5 h of leaching, the Na_2_O content increases to 2.5%.

One of the possible solutions to this problem is double leaching, whereby Al and Si are removed with a partial transfer of goethite to magnetite in the first stage, and the product is then subjected to a ‘curing process’ in a pure alkaline solution to obtain the required degree of blackness in the second stage [27].

### 3.3. Leaching Kinetics

During a fluid–particle heterogenous reaction, a solid particle reacts with a liquid, forming a solid product. If the solid particle that reacts with a liquid shrinks in size, then a shrinking core model can be used to describe the process [44] (p. 566). According to the model, the reaction comprises five steps: diffusion through the liquid film, diffusion through the product layer, the surface chemical reaction, liquid or gas product diffusion through the product layer, and product diffusion through the liquid film to the solution. The slowest step of this process is referred to as the rate-limiting step. Three main models of a shrinking core can be used to describe the process: Equation (13), which describes diffusion through the liquid film; Equation (14), which describes diffusion through the product layer; and Equation (15), which describes the reaction on the surface of the core.

X = kt,(13)

1 − 3(1 − X)^2/3^ + 2(1 − X) = kt, (14)

1 − (1 − X)^1/3^ = kt. (15)

**Figure 8:**
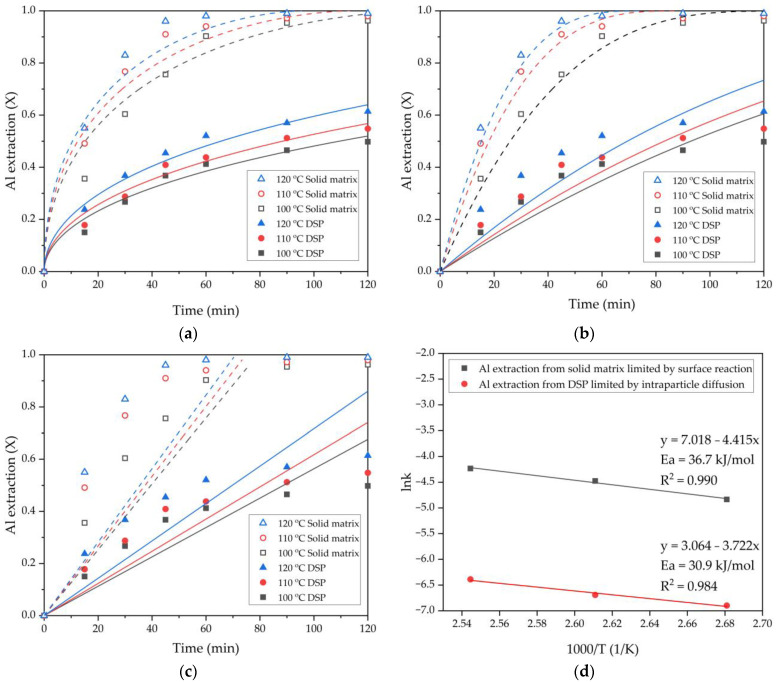
The results of fitting the obtained data for the effect of temperature on the Al extraction from the different phases (points) to Equations (12)–(14) (lines): diffusion through the product layer (**a**); the surface chemical reaction (**b**); diffusion through the liquid film (**c**); the apparent rate constants of the Al extraction from the various phases at different temperatures plotted vs. the inverse temperature (Arrhenius plots) (**d**).

These Equations are valid for the leaching of a solid with a uniform particle size distribution [44]. Therefore, Equations (13)–(15) were used to describe the extraction fraction of Al (X) from the various minerals of the raw sands (Figure 8a–c). Only 2 h leaching time was used in the kinetics calculation because after this time, the fresh DSP began to precipitate (Figure 6). The gibbsite that was not leached during the Bayer process and the Al that can be included in the Al-goethite were denoted as a “solid matrix”, and the Al contained in the DSP was denoted as a “DSP”. The results of fitting the experimental data in Equations (13)–(15) are shown in Table 4; the correlation coefficient values (R^2^) and the corresponding leaching rate constants (k) for all plots in Figure 8a–c are listed. The intraparticle diffusion model (Equation (13)) showed the highest convergence with the extraction of Al from the DSP. Because the DSP has a low leaching efficiency, it can be assumed that the product layer consists of silica-containing minerals that are insoluble under these leaching conditions.

During “solid matrix” phase leaching, there is no new solid product, but the solid matrix of iron minerals, which includes Al-goethite, can play the role of a reaction solid product. However, the model of the surface chemical reaction (Equation (14)) was most suitable to fit the data of Al extraction from the “solid matrix” phase. The Arrhenius plots of lnk versus 1000/T were constructed after the rate constants for both processes were calculated. The activation energy for Al extraction from the “DSP” phase was 30.9 kJ/mol, and for Al extraction from “solid matrix” phase, it was 36.7 kJ/mol. An activation energy greater than 40 kJ/mol is typical for a leaching process limited by the surface reaction [45], confirming that the extraction of Al from a “solid matrix” phase is temperature-dependent and can be limited by the surface reaction.

Figure 8 shows that the degree of Al extraction from a solid matrix of iron-containing minerals is high (almost 100%) using leaching with the transformation of goethite (hematite) to magnetite because goethite must be dissolved before a new solid phase (magnetite) can form [46]. The degree of Al extraction from DSP is relatively low because sodalite is insoluble in alkaline media until a new phase is formed, for example, hydrogarnet [47]. Moreover, after the concentration of silicon in the solution reaches a threshold value, the DSP begins to precipitate again (Figure 6). To enhance the degree of Al extraction from DSP, it is necessary to use several leaching cycles or add lime.

### 3.4. Solid Residue Characterization

#### 3.4.1. Chemical Composition of the Residues

After 2.5 h of leaching at a ferrous sulfate-to-bauxite residue ratio of 1:1 and 120 °C in alkaline media, the alumina extraction ratio was 96.27% for sand and 87.06% for BR. Under these conditions, the Fe content in the sand residue and in the BR residues can be increased to 69.55% and 58.31%, respectively. Table 5 shows the chemical compositions of the solid residues obtained after BR and sands leaching with optimal parameters.

Table 5 shows that the solid residue from sand leaching contains low amounts of impurities: Na and Al contents are less than 0.25%. The content of Si and Ti in this residue is also lower than in traditional bauxite residues. The higher contents of Na, Ti, Si, and Al in the solid residue obtained after BR leaching make it difficult to use this product as a pigment. The high Na content also hinders its use for iron production. Therefore, preliminary leaching with acid or lime causticization should be used to further improve the valorization of BR residues. The P and S contents in these products are less than 0.1% (Table 5), which would be beneficial for iron production.

To evaluate why the difference in the results obtained using BR and sands, the XRD patterns (Figure 3) of the solid residues were investigated. The higher amount of hematite (Al-hematite) in BR (Figure 3) results in the incompleteness of the process (the formation of magnetite from goethite is a faster process from a thermodynamic viewpoint) [48]. Furthermore, there additional sodalite (DSP) in BR that cannot fully dissolve in the alkaline media without lime addition, which leads to increased Na content in the residue. The XRD patterns confirm that the conversion of goethite and hematite to magnetite is more effective for sands. To support this hypothesis, SEM-EDX and Mössbauer analyses of the solid residue were conducted.

**Figure 9:**
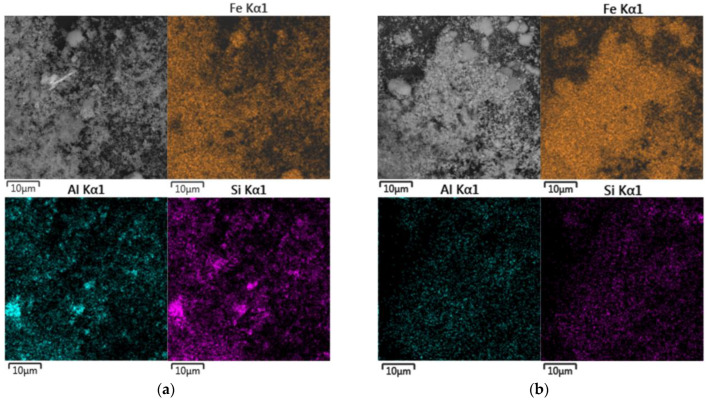

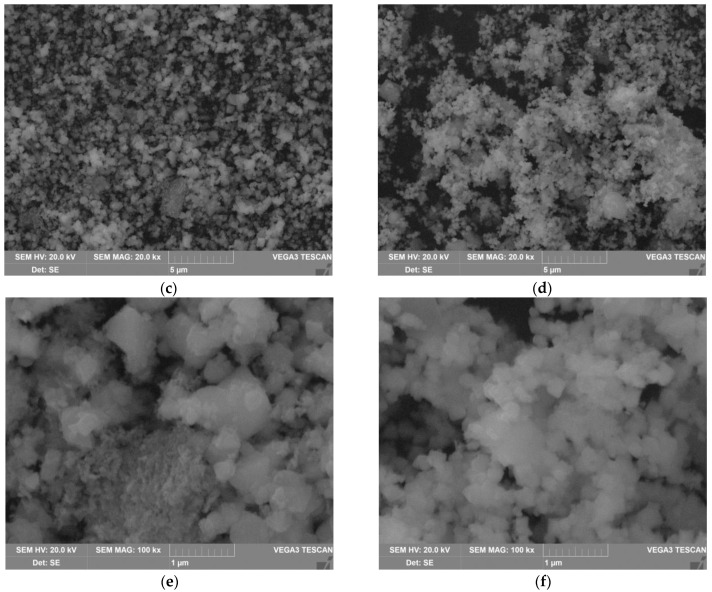
Mapping and morphology of the surfaces of BR and sand residues using SEM-EDX analysis: BSE image and mapping of the BR residue surface (**a**); BSE image and mapping of the sand residue surface (**b**); SEM image of the BR residue surface at 20 kx magnitude (**c**); SEM image of the sand residue surface at 20 kx magnitude (**d**); SEM image of the BR residue surface at 100 kx magnitude (**e**); SEM image of the sand residue surface at 100 kx magnitude (**f**).

According to elemental mapping of the solid residues (Figure 9a,b), the BR solid residue has high Al and Si contents, with lower Fe content, confirming previous observations. Furthermore, the particle size of the sand residue is smaller (see Figure 9c,d), with particles of raw BR visible in Figure 9e.

#### 3.4.2. Mössbauer Analysis

**Figure 10:**
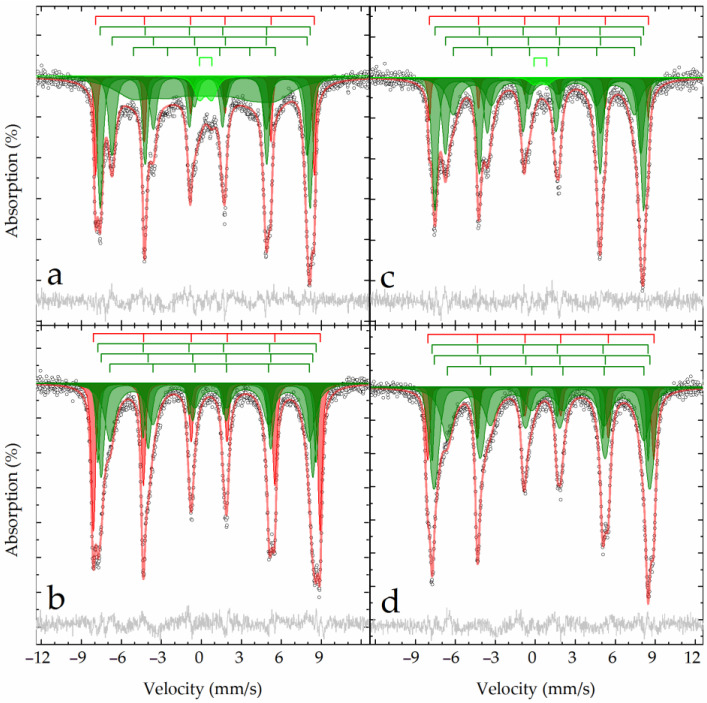
Mössbauer spectra of the BR residue (**a**,**b**) and sands residue (**c**,**d**) samples obtained at 296 K (**a**,**c**) and 77.6 K (**b**,**d**) and their model description according to Table 6.

At room temperature, the Mössbauer spectra of the BR and sand residue samples contain six asymmetric lines of varying widths and intensities, some of which split into two in the region of negative velocities (Figure 10). Furthermore, significant extended absorption is observed in the central part of the spectrum of the BR residue sample. When the samples are cooled to the boiling point of nitrogen, the lines usually narrow, but their splitting and the presence of shoulders indicate that the iron atoms have several crystallographic positions that differ significantly from one another. Experimental spectra can only be satisfactorily described using at least four symmetrical sextets (and one doublet for high-temperature spectra) (Table 6).

The outer sextet with narrow lines belongs to the Al-hematite described above for the raw materials. Moreover, the proportion of Al-hematite in the BR sample was almost one and a half times higher (Table 2); after treatment, the proportion of Al-hematite in the BR residue sample was two times higher compared to the sand residue sample (Table 6). Similarly to the procedure described above, Equation (12) can be used estimate the compositions of Al-hematite as α-(Fe_0.96_Al_0.04_)_2_O_3_ and α-(Fe_0.94_Al_0.06_)_2_O_3_ in the corresponding samples.

The remaining three sextets correspond to iron atoms in different crystallographic positions for nonstoichiometric magnetite of the composition Fe_3-δ_O_4_ ≡ (Fe^3+^)_A_(Fe^2+^_1-3δ_Fe^3+^_1+2δ_#_δ_)_B_O_4_ [49,50]. By analyzing the areas and isomeric shifts of the subspectra related to iron atoms in different crystallographic sites of magnetite, it is possible to estimate the value of the magnetite nonstoichiometric parameter (δ) using Equation (16) [51]:

δ = {Σ(δ_2_ − 3δ_i_ + 2δ_3_) × S_i_ + (δ_2_ − δ_3_)ΣS_j_}/{Σ(3δ_2_ − δ_i_ − 2δ_3_) × S_i_ + 3(δ_2_−δ_3_)ΣS_j_}, (16)

where S_i_ is the relative area of the subspectrum with isomeric shift δ_i_ related to iron atoms at the B site, S_j_ is the relative area of the remaining subspectra, and δ_2_ and δ_3_ are isomeric shifts of iron atoms (+2) and (+3), respectively, in an octahedral oxygen environment for a given temperature (here, δ_2_ = 1.16 ± 0.06 and 1.33 ± 0.09 mm/s for 296 and 78 K, respectively, and δ_3_ = 0.37 ± 0.04 and 0.49 ± 0.04 mm/s for 296 and 78 K, respectively [41]). The results obtained for different temperatures are in agreement with each other, enabling the description of the composition of the samples as Fe_2.75_O_4_ and Fe_2.85_O_4_ for BR residue and sands residue, respectively; the first sample is more oxidized.

**Table 6:** The subspectrum parameters describing experimental Mössbauer spectra obtained at varying temperatures for BR residue and sand residue samples.

Sample			BR Residue						Sand Residue					
Temperature, K	Phase	№	δ	ε (Δ = 2ε)	Γ_exp_	H_eff_	S	δ in Fe_3-δ_O_4_	δ	ε (Δ = 2ε)	Γ_exp_	H_eff_	S	δ in Fe_3-δ_O_4_
			mm/s			kOe	%		mm/s			kOe	%	
296	α-(Fe_1−x_Al_x_)_2_O_3_	1	0.38 ± 0.01	−0.10 ± 0.01	0.28 ± 0.01	508.7 ± 0.2	12.4 ± 0.6		0.38 ± 0.01	−0.11 ± 0.01	0.24 ± 0.01	509.8 ± 0.3	6.1 ± 0.4	
	Fe_3-δ_O_4_	2	0.32 ± 0.01	−0.03 ± 0.01	0.49 ± 0.01	488.4 ± 0.2	29 ± 1	0.15	0.32 ± 0.01	−0.02 ± 0.01	0.52 ± 0.01	485.3 ± 0.2	40.3 ± 0.8	0.25
		3	0.63 ± 0.01	−0.01 ± 0.01	0.62 ± 0.01	454.0 ± 0.2	22.4 ± 0.7		0.62 ± 0.01	−0.03 ± 0.01	0.61 ± 0.02	454.4 ± 0.4	27 ± 2	
		4	0.38 ± 0.01	−0.16 ± 0.03	3.5 ± 0.2	330 ± 4	32 ± 2		0.65 ± 0.01	−0.00 ± 0.01	1.06 ± 0.04	422 ± 2	25 ± 2	
	Fe^3 +^_Oh_	5	0.32 ± 0.02	(0.94 ± 0.03)	0.89 ± 0.07		4.3 ± 0.3		0.38 ± 0.01	(0.94 ± 0.01)	0.9 ± 0.2		1.6 ± 0.2	
77.6	α-(Fe_1−x_Al_x_)_2_O_3_	1	0.50 ± 0.01	−0.09 ± 0.01	0.37 ± 0.01	527.3 ± 0.2	27.4 ± 0.8		0.49 ± 0.01	−0.08 ± 0.01	0.36 ± 0.01	526.0 ± 0.3	12.6 ± 0.9	
	Fe_3-δ_O_4_	2	0.40 ± 0.01	0.03 ± 0.01	0.37 ± 0.01	507.7 ± 0.4	15 ± 1	0.16	0.37 ± 0.01	−0.01 ± 0.01	0.30 ± 0.01	502.6 ± 0.2	10.5 ± 0.8	0.24
		3	0.52 ± 0.01	−0.09 ± 0.01	0.55 ± 0.02	492.5 ± 0.5	26 ± 2		0.52 ± 0.01	−0.03 ± 0.01	0.87 ± 0.02	501.6 ± 0.7	42 ± 1	
		4	0.67 ± 0.01	−0.04 ± 0.01	1.00 ± 0.03	464.2 ± 0.8	32 ± 1		0.84 ± 0.01	−0.07 ± 0.01	1.12 ± 0.03	457.2 ± 0.9	35 ± 1	

δ—isomer shift; ε (Δ)—quadrupole shift (splitting); Γ_exp_—line width; H_eff_—hyperfine magnetic field; S—relative area of a subspectrum №; “δ in Fe_3-δ_O_4_”—magnetite nonstoichiometric parameter [51].

The hyperfine parameters of the minor doublet observed only at room temperature correspond to Fe^3+^ ions in an octahedral oxygen environment [41].

#### 3.4.3. Magnetization

**Figure 11:**
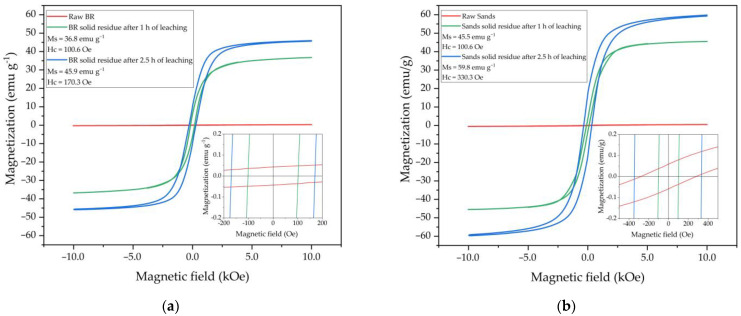
Magnetization curves for the BR and the BR solid residues obtained after BR leaching at 110 °C, C_Na2O_ 360 g L^−1^ for 1 h and 2.5 h (**a**) and the sands and sand solid residues obtained after BR leaching at 110 °C, C_Na2O_ 360 g L^−1^ for 1 h and 2.5 h (**b**).

Figure 11 shows the magnetization measurements of the samples. Both raw samples appear to be magnetically inert. The residue magnetization reaches a saturation value (Ms) of 45–60 emu g^−1^ at a magnetic field of 10 kOe. These values are lower than those of pure synthetic magnetite according to the literature (70–90 emu g^−1^) [52]. However, the values are comparable with those determined for magnetite concentrates obtained from BRs [25]. As shown in Figure 11, the magnetization and the coercive field (H_c_) of both the BR residue and sand residue increases with increasing leaching duration. Additionally, the Ms of the BR residues is lower at all leaching durations, which can be explained by the higher level of impurities in the case of the BR residues.

**Figure 12:**
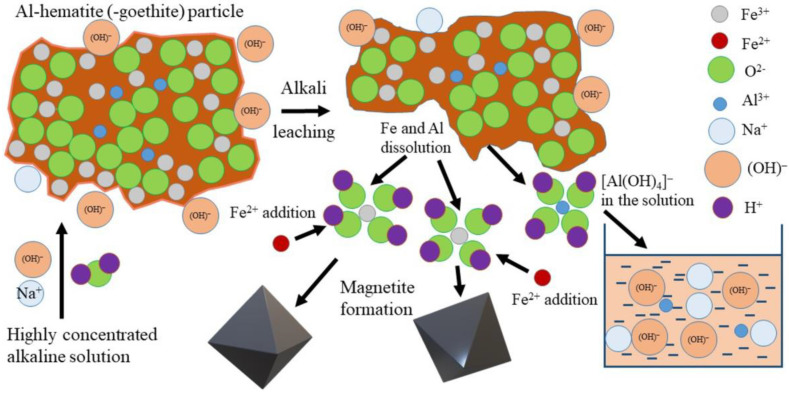
The schematic mechanism of high-iron BR leaching by a highly concentrated NaOH solution in the presence of Fe^2+^.

Considering the results of the experiments and the residue characterization, the following mechanism is suggested for high-iron BR leaching in the presence of Fe^2+^ (Figure 12). The dissolution of iron-containing minerals is limited by the low solubility of iron in an alkaline solution [53]. The presence of Fe^2+^ promotes the extraction of Al from Al-hematite and Al-goethite via precipitation of magnetite from the solution because a new portion of Fe can be further dissolved. Therefore, the accelerative effect is attributed to the magnetization of magnetite and goethite after the minerals are dissolved in a strong alkaline solution.

## 4. Conclusions

In this study, a novel method of treating high-iron bauxite residues from the Friguia alumina refinery by atmospheric pressure leaching in the presence of Fe^2+^ was investigated. According to XRD, TG-DTA, and Mössbauer analyses, aluminum in this type of BR presents as Al-hematite and Al-goethite, which are insoluble in common Bayer leaching. The presence of Fe^2+^ promotes the extraction of Al from Al-hematite and Al-goethite. This effect is due to the magnetization of magnetite and goethite after they are dissolved in a concentrated alkaline solution. The degree of extraction of Al and Fe, Na_2_O content in the residue obtained by alkaline leaching in the presence of Fe^2+^ was analyzed using artificial neural networks and machine learning. The optimal leaching parameters were found to be T = 120 °C, L:S = 10, and τ = 2.5 h, C_Na2O_ = 360 g L^−1^. Under these conditions, the degree of alumina extraction for sand reached 96.27%, and that for bauxite residue reached 86.07%. The grade of iron (total iron in the form of iron elements) in the residue can be increased to 69.55% for sand and 58.31% for BR. The extremely low impurity content in the solid residue obtained after sands leaching and the high blackness make it a viable alternative for pigment production. The difference in extraction degree between sand and BR can be explained by the higher Al-hematite and sodalite contents in BR. Sc acid leaching, lime causticization, or double leaching can be used to reduce the amount of sodalite in BR before it is valorized.

## Figures and Tables

**Table 1:** The chemical compositions of bauxite residue (BR) and sands from the Friguia alumina refinery, Guinea, wt.%.

Sample	Fe	Si	Ti	Al	Na	O	Other
BR	41.70	4.25	4.36	12.34	4.53	30.68	2.14
Sands	56.23	0.77	1.38	5.55	1.26	33.80	1.01

**Table 3:** The results of the semiquantitative calculation of the aluminum-containing phase composition in the BR and sands, wt.%.

Sample	Desilication Product	Aluminum Hydroxide in the Solid Matrix	Reprecipitated Gibbsite
BR	31.3	26.7	42.0
Sands	19.3	80.7	-

**Table 4:** The results of fitting the experimental data (Figure 7) to the shrinking core models.

Equation	Al-Containing Phase	Temperature, °C	Apparent Rate Constant k (min^−1^)	R^2^ (%)
Diffusion through the product layer (Figure 7a)	DSP	100 110 120	0.0010 0.0012 0.0017	0.964 0.972 0.967
	Solid matrix	100 110 120	0.0076 0.0088 0.0105	0.904 0.865 0.800
Surface chemical reaction (Figure 7b)	DSP	100 110 120	0.0022 0.0025 0.0030	0.851 0.861 0.830
	Solid matrix	100 110 120	0.0079 0.0114 0.0145	0.996 0.966 0.971
Diffusion through the liquid film (Figure 7c)	DSP	100 110 120	0.0044 0.0048 0.0055	0.929 0.930 0.912
	Solid matrix	100 110 120	0.0090 0.0093 0.0095	0.870 0.852 0.832

**Table 5:** The chemical compositions of the solid residues obtained after leaching, wt.% (T = 120 °C, τ = 2.5 h, FeSO_4_-to-BR ratio of 1:1).

Sample	Fe	Si	Ti	Al	Na	O	Sc	P	S	Other
BR solid residue	58.31	2.06	3.92	1.33	1.45	31.35	0.006	0.007	0.08	1.58
Sands solid residue	69.55	0.34	1.01	0.15	0.24	27.81	0.005	0.009	0.003	0.90

## Data Availability

Not applicable.
